# Femoral metastasis in previously treated bladder cancer patient: A case report

**DOI:** 10.1002/ccr3.6357

**Published:** 2022-09-14

**Authors:** Tala Jouma Alhejazi, Hassan Bdeiwi, Mohamad W. Sukkari, Mohamad Ibrahim, Ammar Sukari, Hani Alloush

**Affiliations:** ^1^ Faculty of Medicine University of Aleppo Aleppo Syria; ^2^ Department of Oncology Karmanos Cancer Institute, Wayne State University Detroit Michigan USA; ^3^ Department of Orthopedic Surgery Faculty of Medicine, Aleppo University Hospital, University of Aleppo Aleppo Syria

**Keywords:** bladder, bone metastasis, case report, femur metastasis, TCC, transitional carcinoma, urothelial carcinoma

## Abstract

Although treated appropriately, bladder cancer can recur and metastasize. We are reporting the case of a patient with a well‐cured bladder cancer who presented after 14 months with femoral pain which turned out to be a bony metastasis. The patient underwent surgical excision followed by chemotherapy.

## Abstract 2

Secondary bone metastases from a primary transitional carcinoma in the bladder are very rare. In this case, we present a femoral metastasis from a primary urothelial carcinoma in the bladder that had been successfully treated 14 months earlier.

## 1 INTRODUCTION

Bladder cancer forms about 3% of all cancer cases worldwide and 2.1% of cancer deaths.^1^

It is diagnosed more in men than women with a ratio of (3:1). The main risk factor is smoking which is found in about half of affected males and third of affected females. Histologically, it is classified into two major types: transitional cell carcinoma (TCC) and squamous cell carcinoma (SCC). TCC is the most common and mainly caused by aromatic amines. Conversely, SCC is less common and mainly caused by *Schistosoma hematobium*.^2^

Gross hematuria is the main clinical sign of bladder cancer followed by microscopic hematuria.^3^ (70–80) % of cases are in situ tumors, stage Ta and T1. Microscopically, the tumor is papillary in most cases but mixed and solid forms can also be observed. The tumor recurs in about (40–50) % of treated cases.^2^

Bladder cancer has a high potential to metastasize which worsens the overall survival.^4^ It usually metastasizes to lymph nodes, liver, lungs, bones, and peritoneum.^5^

Bone metastases manifest as pain, impaired mobility, and pathological fractures.^6^

Consequently, our case confirms the importance of excluding bone metastasis in bladder carcinoma (BC) if the symptoms mentioned above appear.

## 2 CASE PRESENTATION

A 43‐year‐old smoker, nonalcoholic, male presented to the clinic with pain and movement restriction in his left hip joint. He suffered from a lack of appetite and weight loss.

Two years ago, he was diagnosed with grade III urothelial carcinoma invading the lamina propria and the obtained muscular tissue. He underwent transurethral resection (scrapping the bladder wall) followed by six courses of gemcitabine (Gemzar®) and cisplatin by intravenous infusion, and he was cured.

**FIGURE 1:**
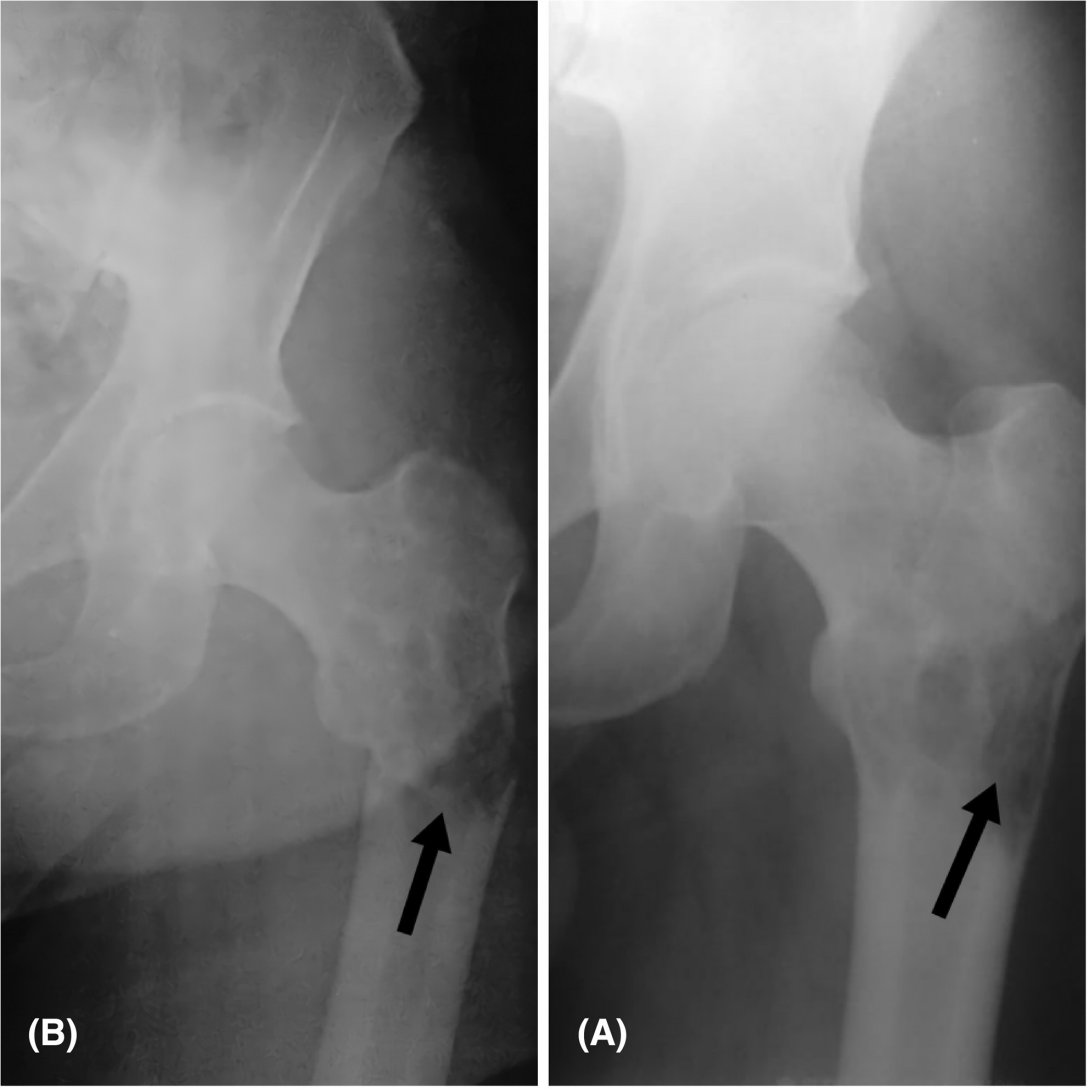
(A) Osteolytic lesion is seen in the left femur before the fracture. (B) The arrow points to the femur fracture

On physical examination, a mild limitation in his hip joint movement was found. Laboratory tests showed elevated ESR, CRP, and ALP with normal CBC and calcium. Hip and thigh X‐Ray showed an area of radiolucency in the subtrochanteric region (Figure 1A). After that, a contrast multi‐slice computed tomography (CT) of the chest, abdomen, and pelvis revealed no pulmonary densities or nodes, no enlarged mediastinal or pulmonary lymph nodes, and no pleural effusion.

**FIGURE 2:**
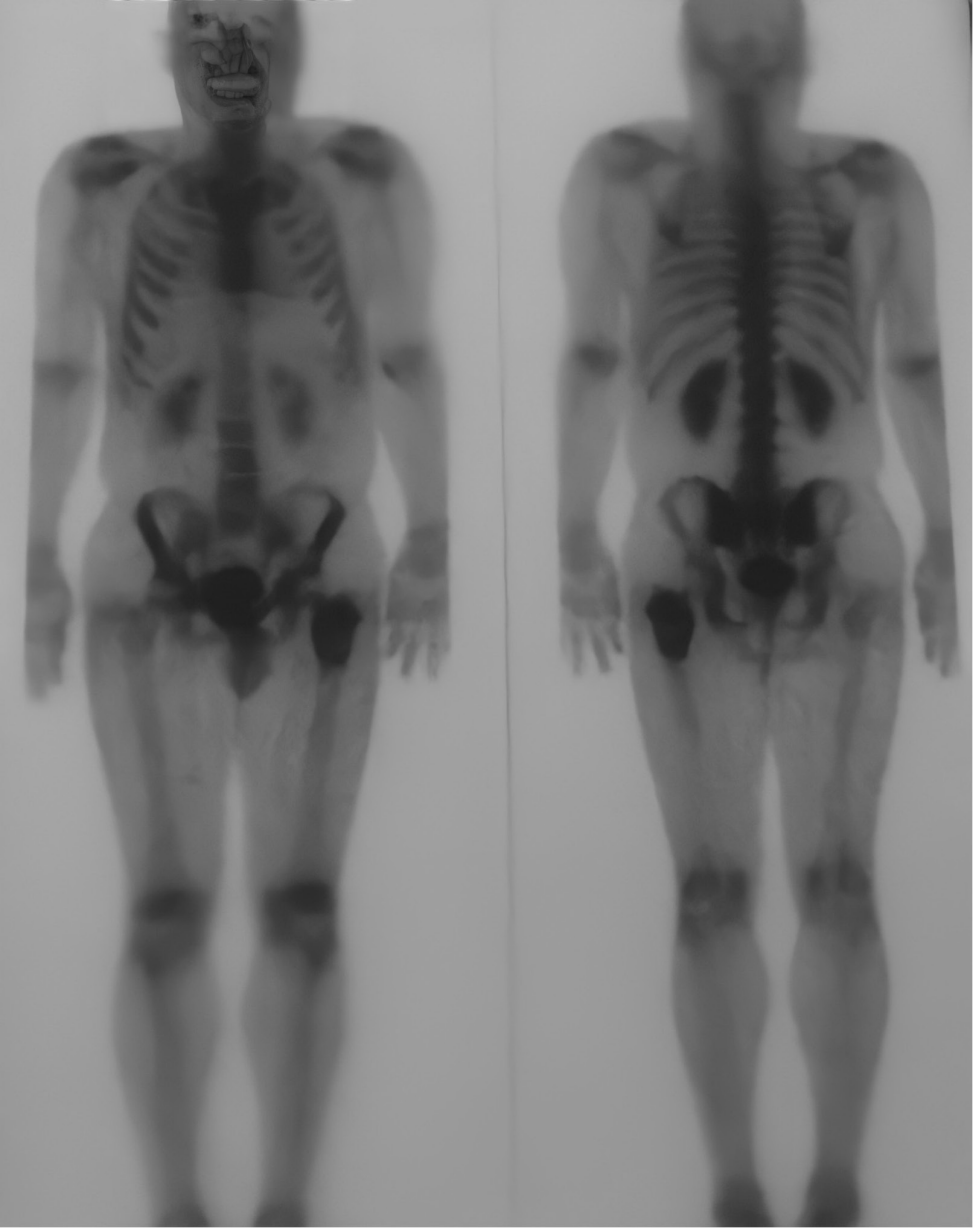
A bone scan showing increased bone metabolism and tracer activity overlying 7.5 cm down the left subtrochantric region

In the abdomen, there was mild liver enlargement with no masses or cysts. The bile duct, adrenal glands, and kidneys were normal.

There was a (60 × 13) mm subserosal mass in the left wall of the bladder. Small lymph node enlargements (<13 mm) were observed around the abdominal aorta and in the pelvis.

After that, the bony CT scan showed vertebral degenerative changes without any osteolytic or osteosclerotic focuses. A bone scan was done following intravenous administration of technetium 99 m MDP, it showed an increased tracer uptake overlying 7.5 cm down the left subtrochanteric region, and the uptake of the resting skeleton was normal so were the kidneys (Figure 2).

**FIGURE 3:**
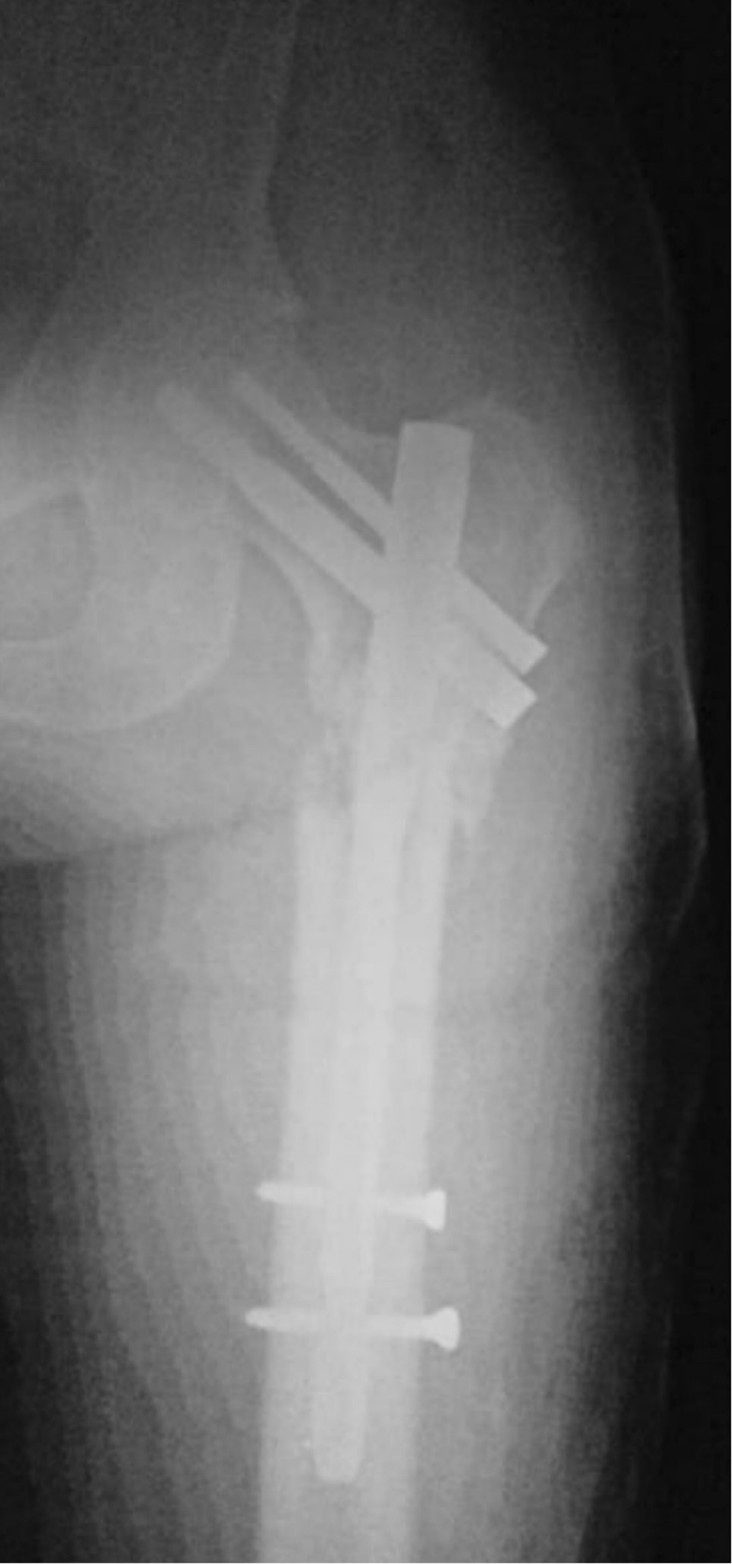
X‐ray image after surgical fixation with PFN and bone cement of the fracture

With this increased bone metabolism in the mentioned area, a secondary neoplastic change was suspected and a biopsy was scheduled to identify the entity of the mass. However, 2 days before the appointment, the patient presented with severe hip pain. The X‐ray showed a subtrochanteric fracture (Figure 1B). He underwent an urgent surgery to remove the mass and to fix the fracture. The bone was fixed with proximal femoral nail (PFN) and bone cement (pmma) (Figure 3).

**FIGURE 4:**
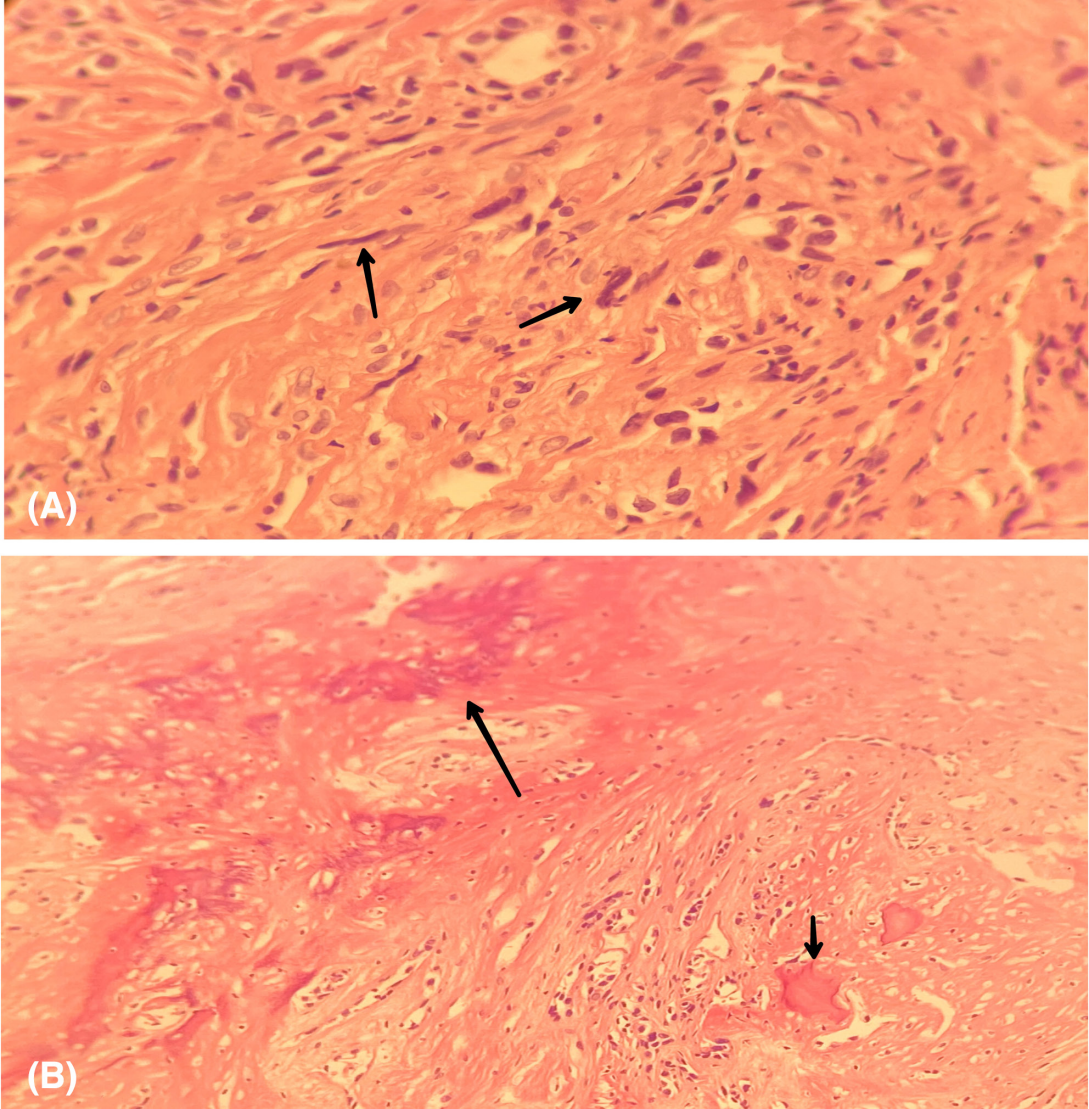
(A) High magnification (100×) histological section of the lesion showing malignant cells which have pleomorphic and hyperchromatic nuclei, with the presence of occasional mitotic figures. (B) Low magnification (40×) fragmented pieces measuring 40 × 25 mm, white in color with bony pieces. The histological changes are those of metastasis of poorly differentiated carcinoma

The pathology report confirmed the metastasis from the previous urothelial cell carcinoma (Figure 4). So, the patient had to be treated again with three courses of vinflunine (Javlor®) and zoledronic acid, one 4‐mg intravenous injection monthly to control the tumor‐induced hypercalcemia. The patient has been placed on a follow‐up program to ensure he does not have any recurrences.

## 3 DISCUSSION

Bladder carcinoma is one of the most prevalent cancers in the world, ranking the 6th most common cancer in the United States, and the 2nd most common genitourinary tumor.^3^ BC usually occurs in older people about the age of 60, with a very varied mortality range from mild to normal and even fatal.^3^ BC occurs mainly in white people, but black people are affected more, particularly men (2:1 men predominance).^3^ The major risk factor for BC is smoking, but bladder stones, urinary tract infections, interstitial cystitis, and presence of catheters were also reported as risk factors.^7^ The most common types of BC are SCC, accounting for 19%, and urothelial carcinoma, also known as TCC accounting for 81%.^7^ The main presentations of TCC are hematuria, dysuria, urgency, and obstructive symptoms.^3^ All patients with the previous signs should be evaluated to exclude TCC by biopsies from different places of the bladder, urine cytology, cystoscopy, and other diagnostic procedures such as fluorescence in situ hybridization, photodynamic diagnosis, and narrow‐band imaging.^8^ CT scan should be done to detect the presence of any metastasis.^8^

After the confirmation of the disease, several treatments can be applied depending on the oncologist's consultation, but usually, the administration of intravesical mitomycin (MMC) or the combination between mitomycin and bacilli Calmette‐Guerin (BCG‐MMC) in carcinoma in situ is the first line in the treatment protocol.^8^ Otherwise, a radical cystectomy must be done for advanced cases which include muscular layer invasion and recurrence cases.^8^ TCC can metastasize to many organs such as the liver, brain, and lungs. Even bone metastases were rarely reported.

Bone metastases are when cancer cells migrate from the original site of cancer and settle in the bones.^9^ They are classified into osteosclerotic and osteolytic lesions according to what the prominent feature is (lysis or sclerosis); this can be determined using radiographic or radiotracers means.^6^ Osteolytic lesions can be found when the source of the lesion is breast cancer, lung cancer, or renal cancer, whereas osteosclerotic ones are found in prostate cancer for instance.^9^ Bone metastases are most common with some types of cancers, especially breast, lung, and prostate. These tumors account for more than 80% of bone metastases.^9^ Most commonly, metastases colonize the axial skeleton (skull, pelvis, ribs) and less commonly the proximal humeri and femora,^9^ lesions of proximal femora are located in the femoral neck (50%), subtrochanteric (30%), and intertrochanteric (20%).^10^ They manifest as pain, impaired mobility, pathological fractures, and hypercalcemia, which leads to kidney and heart failure.^6^ The diagnostic tests include CT, bone scintigraphy, PET‐CT, whole‐body MRI, and CT‐guided biopsy to confirm the diagnosis.^9^ Treatment indications depend on the type and site of the lesion, the response to adjuvant therapy, and overall prognosis.^10^ Non‐surgical treatments include pharmacological agents such as bisphosphonates and denosumab that reduce complications such as fractures, external beam radiotherapy is considered the treatment of choice for uncomplicated lesions that gives (50–80) % pain relief, chemotherapy that depends on the primary tumor can also be used.^6^

Surgical treatment aims to relieve the pain and restore function.^10^ In subtrochanteric and pertrochanteric lesions, intramedullary nails are used, which are load‐sharing devices.^10^ Moreover, the maximum number of interlocking screws—which increase rigidity—and cement usage provide immediate stability.^10^ Lesions in the femur head or neck without acetabular involvement are managed by total arthroplasty or hemi‐arthroplasty.^10^ Lesions in the intertrochanteric area can be managed by either arthroplasty or internal fixation.^10^ Finally, lesions with a lot of bone loss in the subtrochanteric area are managed by endoprosthetic replacement.^10^ Lesions thought that may cause fractures can be prophylactically fixated, and those lesions are determined by Mirel criteria.^10^

Urothelial carcinoma rarely can recur as intertrochanteric metastases after a period of stability and that agrees with our case.

## 4 CONCLUSION

This case highlights the importance of paying close attention to bladder cancer patients even if they were treated successfully as it can recur and even metastasize as in our case. Performing appropriate investigations regularly can be very useful in detecting any recurrence and treating it in its early stages.

## AUTHOR CONTRIBUTIONS

HA has supervised and helped in writing the manuscript. WS, MI, and TA have written the manuscript. HB has contributed to writing and corresponding to this manuscript. AS has reviewed the manuscript.

## FUNDING INFORMATION

The authors declare no source of funding for this manuscript from any organization or any institution.

## CONFLICT OF INTEREST

The authors want to declare that none of them is or was employed by any government agency that has any function other than research and education, and none of them is submitting this manuscript as an official representative or on behalf of the government**.**

## ETHICAL APPROVAL

Not applicable.

## CONSENT

Written informed consent was obtained from the patient to publish this report based on the journal's patient consent policy.

## Data Availability

All data underlying the results are available as part of the article, and no additional source data are required.
